# Comparison of Cone-Beam Computed Tomography and Periapical Radiography in Predicting Treatment Decision for Periapical Lesions: A Clinical Study

**DOI:** 10.1155/2012/920815

**Published:** 2012-09-30

**Authors:** Ashok Balasundaram, Punit Shah, Michael M. Hoen, Michelle A. Wheater, Josef S. Bringas, Arnold Gartner, James R. Geist

**Affiliations:** ^1^Department of Biomedical and Diagnostic Sciences, University of Detroit Mercy School of Dentistry, 2700 Martin Luther King Jr. Boulevard, Detroit, MI 48208, USA; ^2^University of Detroit Mercy School of Dentistry, 2700 Martin Luther King Jr. Boulevard, Detroit, MI 48208, USA; ^3^Department of Endodontics, University of Detroit Mercy School of Dentistry, 2700 Martin Luther King Jr. Boulevard, Detroit, MI 48208, USA

## Abstract

*Objectives*. To compare the ability of endodontists to determine the size of apical pathological lesions and select the most appropriate choice of treatment based on lesions' projected image characteristics using 2 D and 3 D images. *Study Design*. Twenty-four subjects were selected. Radiographic examination of symptomatic study teeth with an intraoral periapical radiograph revealed periapical lesions equal to or greater than 3 mm in the greatest diameter. Cone-beam Computed tomography (CBCT) images were made of the involved teeth after the intraoral periapical radiograph confirmed the size of lesion to be equal to greater than 3 mm. Six observers (endodontists) viewed both the periapical and CBCT images. Upon viewing each of the images from the two imaging modalities, observers (1) measured lesion size and (2) made decisions on treatment based on each radiograph. Chi-square test was used to look for differences in the choice of treatment among observers. *Results*. No significant difference was noted in the treatment plan selected by observers using the two modalities (*χ* ^2^(3) = .036, *P* > 0.05). *Conclusion*. Lesion size and choice of treatment of periapical lesions based on CBCT radiographs do not change significantly from those made on the basis of 2 D radiographs.

## 1. Introduction

The clinical, radiographic, and histological diagnosis of periapical lesions has been a challenge [1, 2]. Current diagnostic methods help in fair assessment of accurate size and nature of a periapical lesion which determine the treatment and prognosis of the tooth in question [3–5]. Making the correct pulpal and periapical diagnosis is helpful for treatment and prognosis of the tooth [1]. Even if biopsy is the gold standard in differentiating granulomas from cysts, clinicians are aware of the difficulty in obtaining biopsies in routine clinical practice [6, 7]. Therefore, there is an immediate need for a noninvasive method to diagnose lesions involving the periapical area. 

Radiographs play an important role in the success or failure of endodontic treatment. This could be due to fact that interpretation of images which influences success or failure also depends on several factors, including the clinician's experience in interpreting images. However, due to inherent drawbacks such as superimposition and distortion, it is sometimes difficult to detect periapical (PA) lesions on two-dimensional images. Two-dimensional radiographs can image PA lesions to be detected only when bone mineral loss reaches 30%–50% due to structured noise of superimposed cortices [8]. Therefore, only 50–55% of small and medium sized lesions can be diagnosed as periapical disease [9].

Reports indicate that CBCT images provide clinically relevant information not found in periapical images [10–13]. A recent study used CBCT as the reference imaging modality (gold standard) to compare the accuracy of periapical and panoramic radiography in detecting periapical lesions. This study concluded that CBCT had better diagnostic accuracy than periapical and panoramic radiography. Panoramic images were the least sensitive in detecting lesions. The question that is valid to ask now is, does evaluation of a periapical lesion with CBCT change the estimation of size and choice of treatment among endodontists compared to periapical radiography [14]? Selecting the most appropriate choice of treatment using the most accurate imaging modality would ultimately reduce cost and morbidity significantly in patients undergoing endodontic therapy [15]. 

The purpose of this study was to compare lesion size and choice of treatment relative to the available radiographic information from periapical radiography and CBCT. The null hypothesis is that there is no difference in the lesion size and choice of treatment of periapical lesions between conventional periapical radiographs and CBCT images.

## 2. Materials and Methods

### 2.1. Study: Population/Sample

The study group comprised twenty-four subjects, 11 women, and 13 men, with an average age of 53 years (range 18–88 years) Table 1. Approximately one hundred subjects were examined to achieve a study sample of twenty-four. All subjects reported to the Endodontic Division of the University of Detroit Mercy School of Dentistry with symptoms suggestive of a periapical lesion. Subjects were recruited consecutively during the period from March 2010 to December 2010. History of present illness and clinical examination (including palpation, percussion, and cold test) of the tooth involved was performed before any radiographic procedure was undertaken. Subjects were recruited to the study  only after the initial intraoral periapical radiograph showed a periapical lesion of size greater than 3 mm. Both single rooted and multirooted teeth with periapical lesion size equal to or greater than 3 mm on intraoral periapical radiography were included in the study. Only one tooth from each patient selected was used for the study. If a patient had more than one tooth that qualified for the study, the tooth with the most severe symptoms and maximum size of the periapical lesion as determined by conventional radiography was selected. Previously root-treated teeth and teeth with restorations were also included in the study. Exclusion criteria included those teeth that needed immediate therapy due to an endodontic emergency. Endodontically involved teeth that had history of trauma or radiographic evidence of fracture were excluded from the study. No specific control group was used for either of the radiographic modalities tested since teeth other than the study tooth in the same jaw were also imaged and served as internal controls. The internal control tooth was scanned using the same CBCT scanning protocol. The study was reviewed and approved by the University of Detroit Mercy Institutional Review Board (UDM IRB Protocol no. 0910-34).

### 2.2. Image Acquisition

#### 2.2.1. Conventional Radiographs

**Figure 1:**
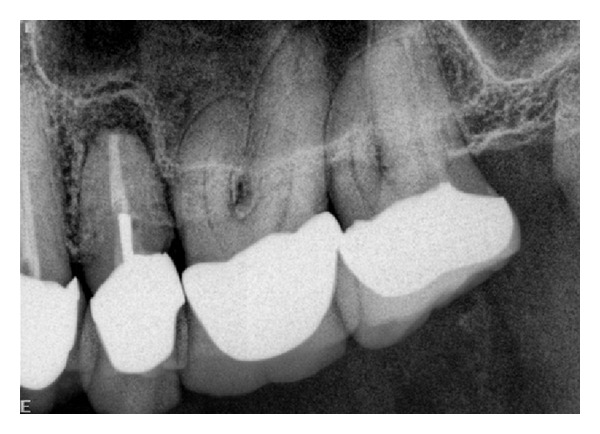
Intraoral periapical radiograph of second premolar tooth used in the study.

Two-dimensional radiographs (intraoral periapical) were obtained with an intraoral dental X-ray machine (Planmeca Intra, Planmeca USA Inc, IL, USA) using the Paralleling Axis Technique and round collimator (2.86′′ diameter) with variable kVp and mAs and focal spot size 0.7 mm × 0.7  mm. Schick CMOS sensors (Schick Technologies Inc., Long Island City, NY, USA) were used to record the images. Schick CDR software was used to display images in real-time on a monitor. The images were stored as 8-bit TIFF (Tagged Image File Format) files at maximum quality (100%) (see Figure 1). All images were approved for adequate image quality by the principal investigator. Adequate image quality essential for diagnosis was established in a subjective manner after comparing previous images obtained from this radiographic modality and standardizedwith the ones that would be used for this study. The size of the periapical lesions was measured with a measurement tool available in the Schick software. Teeth with periapical lesions equal to or greater than 3 mm as seen on the two-dimensional radiographs were further recommended for a CBCT scan. Dimensional calibration for size was applied to the two-dimensional images upon export to the study software. Calibration was applied using the calibration tool in the software in which all the images were observed. Calibration of images was achieved through a technique as follows. A particular distance was measured on the periapical image obtained upon exposure, and the same distance was measured when this image was exported to the software through which the images were available for the observers to view.

#### 2.2.2. Cone-Beam Computed Tomography (CBCT) Scan

**Figure 2:**
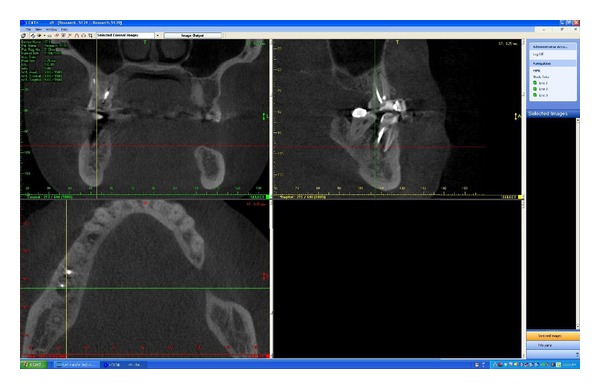
Cone Beam CT image of molar tooth as viewed by observers in the study.

After explaining the CBCT scan procedure and obtaining approval from the patient for the imaging procedure, a CBCT scan of the study patient was acquired using the 17–19 Platinum Next Generation I-CAT CBCT scanner (Imaging Sciences International, Hatfield, PA, USA) and standard scanning protocols (pulsed exposure, 120 kVp, 3 to 7 mA, 14.7 second exposure time, 0.25 × 0.25 × 0.25 mm isotropic voxel size, 14 bit, maxilla or mandible) (see Figure 2). Collimation was fully adjusted to include the maxilla or mandible only. A limited (maxilla or mandible) FOV (field of view) was used to scan the tooth involved. The FOV for the maxilla and mandible was 16 cm (*d*) × 6 cm (*h*). A Cesium Iodide flat panel detector was used to acquire images. Only the arch (maxillary/mandibular) with the tooth and associated periapical lesion was scanned. The effective radiation dose varied from patient to patient. All pertinent patient information on the scanned images was anonymized prior to being viewed by observers.

### 2.3. Image Viewing

Scan data of subjects was exported from proprietary i-CAT vision software (i-CAT vision Q, Imaging Sciences International, Hatfield, PA, USA) to a previous version of i-CAT software in.xstd format. Images were examined with this specific proprietary I-CAT software. This helped anonymize patient data on the images to be viewed by observers. CDR Schick PA images (Schick Technologies, Inc. Long Island City, NY, USA) were exported as TIFF files. Images from both modalities were then imported into an interface viewing software, XV lite software (Apertyx Inc., Akron, OH, USA). (Figure 1: intraoral periapical radiograph, Figure 2: CBCT image as viewed by observers) The two-dimensional images were spatially calibrated according to known dimensions of the native images. Periapical images were viewed in an office on a Dell UltraSharp 2407WFP 24-inch Widescreen Flat Panel LCD monitor (Dell Inc., Round Rock, Texas, USA), running under Microsoft Windows XP professional SP-1 (Microsoft Corp, Redmond, WA, USA). The monitor specifications include a native resolution of 1920 × 1200 at 60 Hz, pixel pitch of 0.27 mm, brightness of 450 cd/m^2^, and contrast ratio of 1000 : 1. The CBCT images were displayed on a monitor at the CBCT workstation. The same monitor was not used for viewing both PA and CBCT images for accession purposes. It was easier for observers to view CBCT images at a monitor attached to the CBCT scan acquisition hardware. The PA images were viewed by the observers at the principal investigator's office as these images could be easily exported from the Endodontic Department upon capture to that office. All images were viewed under acceptable room lighting conditions.

Six endodontists with varying levels of expertise were selected as observers. The observers included both novice and experts. Three endodontists were senior faculty in the Department of Endodontics and three of them were junior part-time faculty. The observers were calibrated through a training session. The observers were asked to perform the following with the two imaging modalities separately: (a) measure the extent of the periapical lesion (greatest distance (diameter) in millimeters and (b) choose treatment for particular tooth (root canal treatment, periapical surgery, root canal treatment and periapical surgery and no endodontic treatment). The observations were performed in two separate sessions: one for PA images and another for CBCT images. Images from each modality were viewed only once by each observer. This was done to enable observers could score all images between the two modalities in a timely fashion. The observation sessions were separated by two weeks between the two modalities. The presentation of the images to the observers among sessions was randomized. All observers examined 24 images acquired from 24 different teeth (24 subjects) from the two imaging modalities. They measured the lesion size and scored their choice of treatment for each tooth. Overall, a total of 288 scores were that is, 6 observers x obtained (24 images for PA + 24 images for CBCT) each for lesion measurement and treatment choice.

### 2.4. Data Analysis

Mann-Whitney *U* test was used to assess for differences in the measurement of lesion sizes by all observers among the two modalities (*P* > 0.05). Wilcoxon signed ranks test was used to look for differences in the measurement of lesion sizes among the two modalities by each observer. Intraclass correlation coefficient was calculated among observers for each modality with respect to lesion size and choice of treatment.

Chi-square test was done to look for differences in the choice of treatment selected by all observers for the two modalities. The method of Bland and Altman was used to assess observer agreement of selected treatment between the two imaging modalities. 

## 3. Results

**Figure 3:**
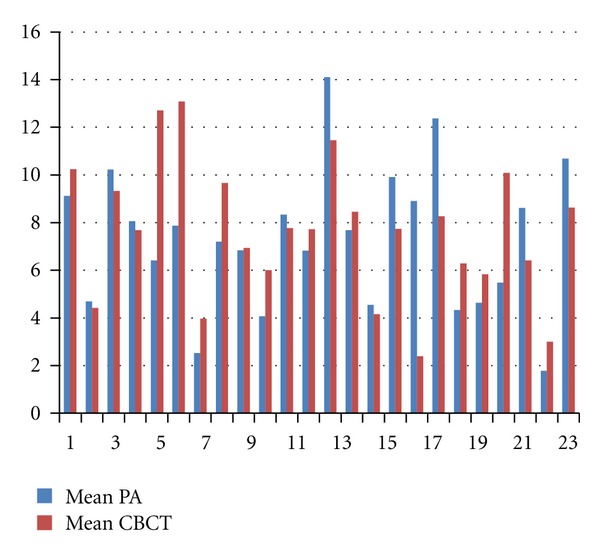
Mean of all lesion size measurements of 24 teeth by observers (6) comparing PA and CBCT. *x*-axis: number (*n*) of images, *y*-axis = lesion measurement (mm).

The mean for lesion sizes among the two modalities for the study sample is shown in Figure 3. The 95% confidence interval for either the lower or upper limit of agreement was less than two standard deviations (2SD = 2∗2.86 = 5.72). Table 2 shows no statistically significant difference between the two modalities in terms of the lesion size measured by individual observers (Mann-Whitney *U* = 9720.500,  *Z* = −.916,  *P* > 0.05). The intraclass correlation coefficient for the observers was 0.465 for treatment decision, and the high agreement (0.947) for lesion size.

**Figure 4:**
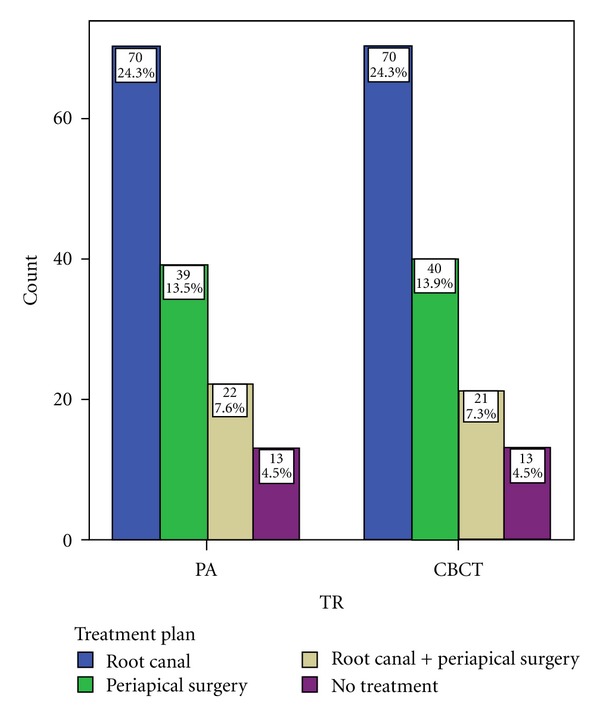
Bar graph showing treatment decisions between the two modalities (PA and CBCT).

Chi-square test revealed no significant difference in the treatment plan selected by observers between the two modalities (*χ* ^2^(3) = .036, *P* > 0.05). For some treatment decisions, there was equal or almost equal selection between the two modalities.  Table 3, Figure 4.

**Figure 5:**
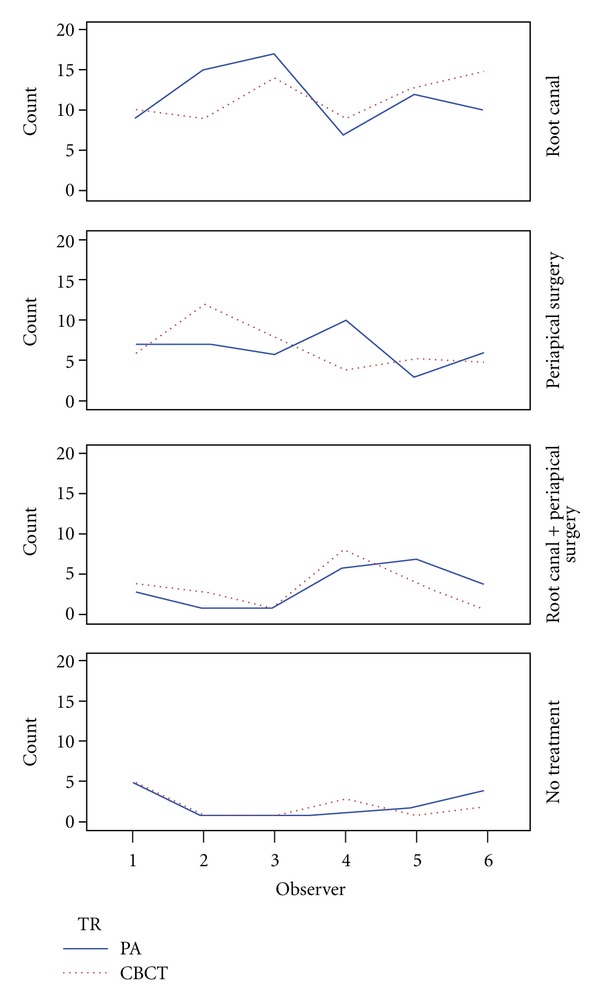
Graph showing trend among observers in selecting treatment decisions among the imaging modalities (PA and CBCT).

Figure 5 shows trend among observers in selecting treatments between the two modalities. The 95% confidence interval for either the lower or upper limit of agreement was less than two standard deviations (2SD = 2∗2.86 = 5.72). This implies that the chance that observers chose different treatments from the two imaging modalities is minimal.

**Figure 6:**
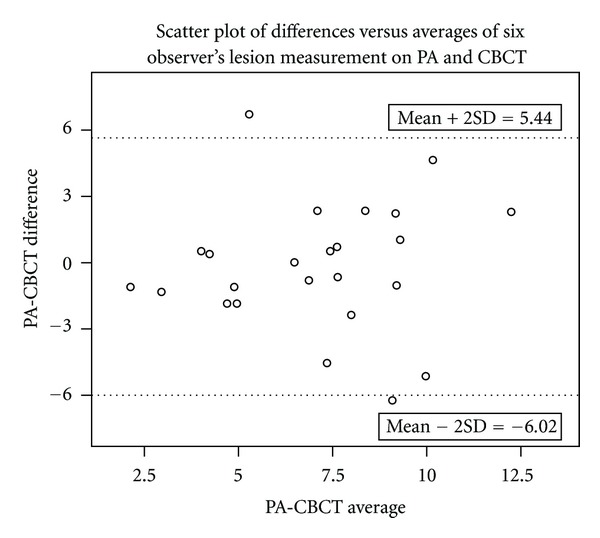
Scatter plot of differences versus averages of six observer's lesion measurement of PA and CBCT (in mm).

All observers were accurate in identifying the lesion in the study tooth. The intraclass correlation among observers for each modality and both the imaging modalities together with reference to treatment selection and lesion size was calculated. (Tables 4 and 5). Though there was no statistical significance between the two modalities in terms of lesion size measured by individual observers, there was appreciable variability when the lesion size measurements of all the six observers were averaged (Figure 6).

## 4. Discussion

With the introduction of cone beam CT in dentistry, several applications of this advanced imaging modality, including endodontics, are being investigated. In endodontics, the use of CBCT involves diagnosis of pathosis, treatment planning, and diagnosis of trauma to dentoalveolar structures and evaluation of previously root-treated teeth, especially for missed canals [16–19]. 

Even though the benefits of CBCT in endodontics are well understood, this particular study was undertaken to assess if the visualization of the third dimension as seen in CBCT images would alter the choice of treatment made by endodontists. No difference in treatment plan was noticed between the two imaging modalities. It is also interesting that the number of various treatment decisions (root canal treatment, periapical surgery, root canal treatment plus periapical surgery, and no endodontic treatment) was equal between the two imaging modalities. This led investigators of this study to believe that although CBCT images show “more” information, this additional information would not necessarily translate into selection of a treatment different from the decisions made with the periapical images.

In our study, six endodontists viewed images from both modalities (PA and CBCT) and made their treatment decisions. The intraclass correlation with regards to the treatment decision was only moderate (0.465). This could be attributed to the varying clinical experience levels (novice to highly skilled). Previous studies indicate that the long-term stability of observers in detecting periapical radiolucencies on conventional radiographs was satisfactory [20]. The observers in our study differed in their expertise with interpretation of CBCT images. Two observers were well versed in interpreting CBCT images and were using this technology on a regular basis while others had only knowledge of the CBCT technology but were not well experienced in interpreting images. With reference to lesion size, there was, however, a high level of agreement (0.947) among observers between the two imaging techniques. This finding is in accordance with similar studies which confirm that periapical lesion measurements (when adequately calibrated) are the same between CBCT images and traditional two-dimensional radiographic images [21].

It is important to analyze the difference in radiation doses from the two imaging modalities under investigation in this study. Several studies have assessed the effective radiation dose from different CBCT scanners available in the market [22]. Comparisons in radiation dose between medical CT and CBCT have also been made through scientific studies [23, 24]. The mean organ dose to organs such as the brain, thyroid, and parotid gland becomes extremely important while performing a CBCT scan [25]. CBCT doses also differ with reference to the particular scanning protocol that is being selected for a specific study. Short scan times and standard resolution (not high resolution) scan protocols would reduce the radiation doses from these procedures [26]. The potential risk to the patient due to this increase in radiation dose can be minimized by using a limited field of view (FOV). Newer CBCT scanners customized specially for endodontic purposes have the ability to scan only the particular tooth in question. However, in our study, the CBCT scanner utilized did not have the ability to scan only the tooth and the entire jaw (maxilla/mandible) had to be imaged. The effective dose using the 2007 ICRP (International Council on Radiation Protection) calculation for the scanner and the protocols used in this study varies from 40 to 69 *μ*Sv [27]. The effective dose from the Next Generation CBCT scanner used in this study is comparable to the dose from newer CBCT machines currently being marketed only for endodontic purposes, for example Kodak 9000, with an effective dose of 21 *μ*Sv for a 4 × 5 cm jaw sextant. Also, we used a scan protocol with an option to obtain higher resolution images since there was a need to display the best quality images for the observers to view [28]. The scan resolution used in this study is similar to other studies of this nature. Also lesion size differences between the two imaging modalities can be compared only when highly resolvable images are available.

This study is not without its limitations: (i) the study sample (*n* = 24) is relatively small and it is not known if a larger sample would have produced a different outcome, (ii) history and clinical information (signs and symptoms) of study subjects were not provided to the observers at the time of the viewing sessions, so it is not certain if this information would have produced a significant difference in treatment selection between the two imaging modalities, (iii) the clinical experience of observers varied from novice to most skilled, (iv) the radiation dose from the CBCT imaging procedure in the presence of an already existing radiographic procedure (periapical radiographs) should be validated, and a separate study comparing the effective dose of radiation used in CBCT imaging and conventional radiography for endodontic purposes is required, and (v) it is not known if a different resolution of CBCT images would have affected the results since native resolution of CBCT images was used by observers with no image enhancements (other than brightness and contrast) being performed  for either modalities.

It is now well recognized that prospects for the use of CBCT in endodontics in the future appear promising. However, there are several factors such as diagnostic yield, radiation dose and selection criteria to be considered before this technology becomes incorporated into the diagnostic armamentarium in endodontics [29]. Since interpretation of CBCT images requires additional training, care must be taken to avoid misinterpretation by clinicians who have not had the required training. For example, with an untrained eye, artifacts such as beam hardening due to metallic restorations on CBCT images could be misdiagnosed as a carious lesion. Endodontists aspiring to use this technology should either train themselves or seek the help of a maxillofacial radiologist who can interpret the images for them. Also, when CBCT images do not offer additional clues to diagnosis, periapical radiographs should be referred for more information. Therefore, the notion that CBCT would replace conventional periapical radiographs is far-fetched. Another factor to be considered is the viability and cost-effectiveness of using CBCT in a clinical setting. Not all endodontic offices currently include CBCT in their diagnostic work up. At the present time, the request for CBCT scans for endodontic purposes is not uniform between specialists for the same reason. The cost of a CBCT scan is several fold compared to a conventional periapical radiograph and the increase in cost needs to be justified. 

In conclusion, the null hypothesis proposed in our study was accepted. It was determined from our study that the choice of treatment in endodontics does not change significantly even with the inclusion of an advanced imaging modality such as cone beam CT. The results of this study, however, should be interpreted with caution. Although CBCT performed almost the same as periapical radiography, the potential advantages of CBCT in other areas of endodontics including assessing proximity of teeth to anatomic structures, such as mandibular canal and maxillary sinus, cannot be underestimated. This study also proves the robust diagnostic potentials of traditional two-dimensional imaging modalities. Further studies with a larger sample size are underway to further confirm the results obtained from this study and identify appropriate areas in endodontics where CBCT imaging would play a vital role.

## Figures and Tables

**Table 1:** Study demographics including tooth no., patient age, and gender.

#	Tooth no.	Age	Sex
1	2	53	F
2	19	60	F
3	30	38	F
4	19	59	F
5	7	55	M
6	12	74	M
7	31	44	M
8	18	41	M
9	7	69	F
10	21	60	F
11	28	88	M
12	14	56	M
13	10	33	M
14	9 and 10	21	M
15	3	77	M
16	8	18	M
17	30	38	F
18	18	52	F
19	2	45	M
20	13	70	M
21	15	55	F
22	9	62	M
23	13	52	F
24	31	53	F

**Table 2:** Mann-Whitney *U* test comparing PA versus CBCT for lesion sizes across 6 observers.

				Monte Carlo Sig. (2-tailed)		
Mann-Whitney *U *	Wilcoxon W	*Z*	*p* (2-tailed)	Lower	Upper	Sig.
9720.50	20160.50	−0.92	0.360	0.353	0.377	0.365

**Table 3:** Chi-square test to look for differences in treatment plan between PA and CBCT.

	Chi-square tests		
	Value	df	Asymp. Sig. (2-sided)
Pearson chi-square	.036^a^	3	.998
Likelihood ratio	.036	3	.998
Fisher's exact test	.074		
Linear-by-linear	.004^c^	1	.952
Association			
*N* of valid cases	288		

*χ* ^2^(3) = .036, (*P* >0.05).

**Table 4:** Intraclass correlation among observers for PA and CBCT with reference to treatment selection.

Modality	Avg. measures	95% Confidence		*F* test with true			
		Interval		Value 0			
		Lower bound	Upper bound	Value	df1	df2	Sig
PA	0.435	0.002	0.723	1.771	23	120	0.025
CBCT	0.378	0.103	0.695	1.608	23	120	0.053
PA + CBCT	0.465	0.086	0.733	1870	23	264	0.011

**Table 5:** Intraclass correlation among observers for PA and CBCT with reference to lesion size.

Modality	Avg. measures	95% Confidence		*F* test with true			
		Interval		Value 0			
		Lower bound	Upper bound	Value	df1	df2	Sig
PA	0.951	0.913	0.976	20.346	23	120	0
CBCT	0.965	0.938	0.983	28.742	23	120	0
PA + CBCT	0.947	0.909	0.973	18.834	23	264	0
